# Phosphoenolpyruvate Carboxykinase Maintains Glycolysis-driven Growth in Drosophila Tumors

**DOI:** 10.1038/s41598-017-11613-2

**Published:** 2017-09-14

**Authors:** Rashid Hussain, Zeeshan Shaukat, Mahwish Khan, Robert Saint, Stephen L. Gregory

**Affiliations:** 1 Department of Genetics and Evolution, University of Adelaide, Adelaide, 5006 Australia; 2 Flinders University, Adelaide, 5042 Australia

## Abstract

Tumors frequently fail to pass on all their chromosomes correctly during cell division, and this chromosomal instability (CIN) causes irregular aneuploidy and oxidative stress in cancer cells. Our objective was to test knockdowns of metabolic enzymes in *Drosophila* to find interventions that could exploit the differences between normal and CIN cells to block CIN tumor growth without harming the host animal. We found that depleting by RNAi or feeding the host inhibitors against phosphoenolpyruvate carboxykinase (PEPCK) was able to block the growth of CIN tissue in a *brat* tumor explant model. Increasing NAD+ or oxidising cytoplasmic NADH was able to rescue the growth of PEPCK depleted tumors, suggesting a problem in clearing cytoplasmic NADH. Consistent with this, blocking the glycerol-3-phosphate shuttle blocked tumor growth, as well as lowering ROS levels. This work suggests that proliferating CIN cells are particularly vulnerable to inhibition of PEPCK, or its metabolic network, because of their compromised redox status.

## Introduction

Chromosomal instability (CIN) refers to cell divisions that cannot maintain chromosomal integrity or number. This can be caused by defects including elevated DNA damage, weakened cell cycle checkpoints or an aberrant mitotic spindle^1^. CIN is a common phenotype of human tumours and generates genetic variation that has been associated with tumour evolution, the development of drug resistance and the consequent poor prognosis of CIN cancer patients^2^. We and others have proposed that CIN itself could be an attractive target for chemotherapy, as it is a relatively cancer-specific phenotype ^3–6^. However, as CIN cells are necessarily genetically diverse, it is challenging to identify conserved features of CIN cells as potential targets. Our approach has been to induce CIN in a genetically uniform population of cells *in vivo* in *Drosophila* and to screen for genes that can be knocked down to kill CIN cells without affecting normal proliferating cells^3, 7, 8^. We hypothesize that the candidates giving widespread cell death in CIN cell populations are targeting vulnerabilities common to a wide range of aneuploidies.

This approach identified plausible targets such as JNK signalling and centrosomal regulators that could be depleted to give CIN-specific lethality^3^. In addition, we found metabolic targets such as Phosphoenol pyruvate carboxykinase (PEPCK), and Glucose-6-phosphate dehydrogenase (G6PD)^7^. Knockdown of these genes gave increased mitochondrial output, reactive oxygen species (ROS), DNA damage and cell death in CIN cells without affecting normal proliferating cells. Tumours are often metabolically unlike their surroundings, with elevated glycolysis for anabolism rather than ATP generation^9^. This metabolic demand is shared to some extent by all proliferating cells, as they must generate cellular building blocks before they can replicate their DNA and divide. CIN tumours carry an additional burden, however, as it has been observed that aneuploid cells suffer redox stress in proportion to their aneuploidy^10^. Though we lack a detailed understanding of how aneuploidy causes redox stress, the evidence implicates a combination of elevated ROS levels and protein turnover problems^11^. The combination of this redox stress and a Warburg metabolism makes CIN tumours potentially vulnerable to metabolic intervention that does not affect normal cells.

Having found metabolic targets that were able to kill proliferating cells with induced CIN, we wished to understand their mechanism of action in the context of a growing CIN tumour. In this paper we describe the CIN status of *brat* explant tumours and their use as a fly CIN tumour model. Chemical as well as genetic inhibition of metabolic candidates in this model identified targets such as PEPCK that could effectively block tumour growth. Elevated levels of ROS were observed in the targeted tissue, and adding antioxidants could rescue growth. Experiments to identify the source of the ROS suggested that pressure to clear cytoplasmic NADH generated in glycolysis was leading to ROS generation by the glycerol phosphate shuttle. Our results suggest that metabolic interventions that constrain clearance of NADH can generate toxic ROS levels in CIN tumours without harming the host.

## Results

### Characterization of *brat*^*RNAi*^ as a CIN tumor model

**Figure 1:**
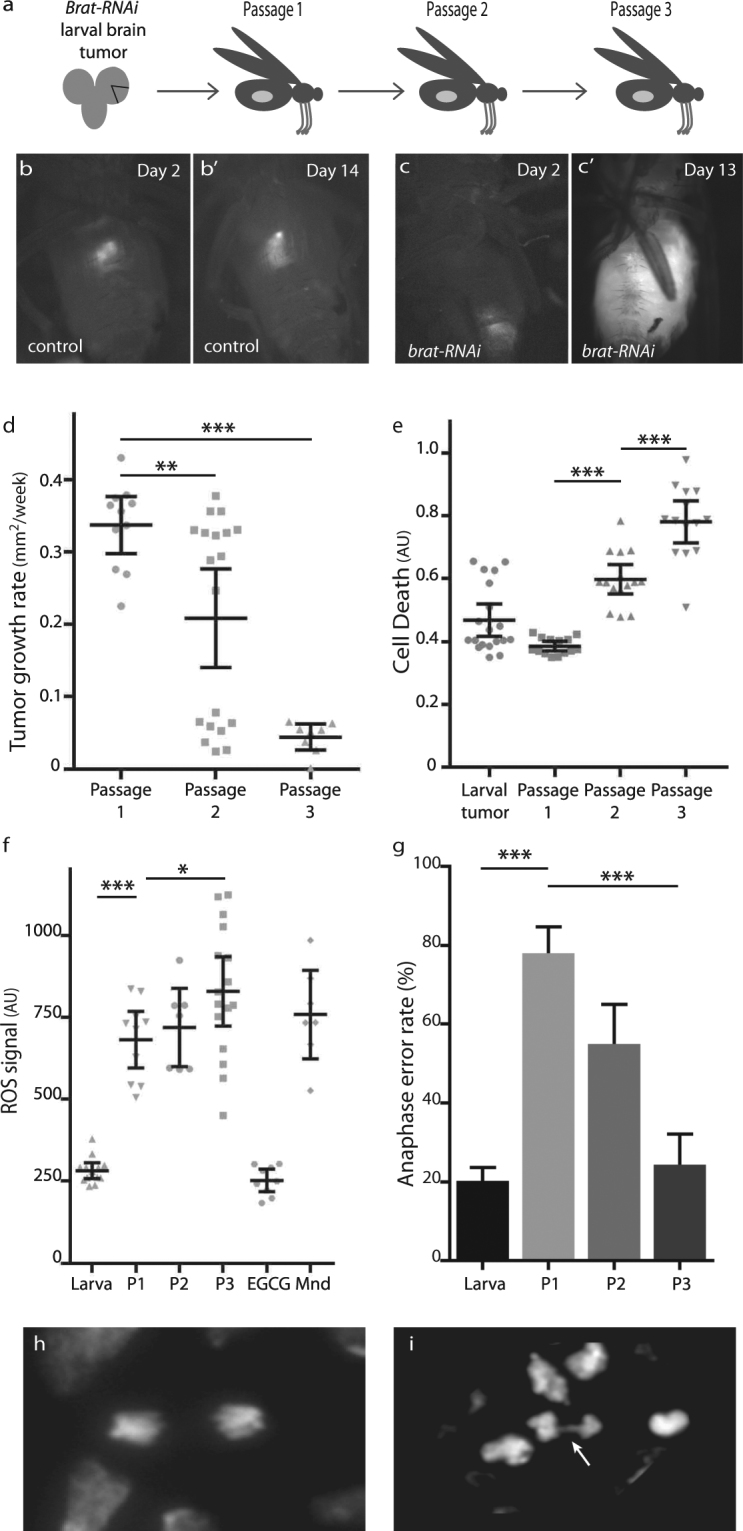
Characterization of *brat-RNAi* as a CIN tumor model. (**a**) RFP-labelled 3rd instar larval brain tissue depleted for Brat (*da* > *Gal4*; UAS-RFP; UAS-*brat*-RNAi) was dissected and transplanted into the abdomen of wild type adult hosts. Serial passages of the tumor explant were carried out into a new host within a fortnight to allow the tumor growth to be continued. (**b**) Control 3rd instar larval brain tissue (*da* > Gal4; UAS-RFP) was transplanted and showed no growth after 2 (**b**) or 14 days (b’). Labelled 3rd instar larval brain tissue explants depleted for Brat (*da* > *Gal4*; UAS-RFP; UAS-*brat*-RNAi) showed considerable growth on day 13 (**c**) compared to day 2 (**c**) after transplantation. (**d**) The average growth rate of *brat* tumor explants (*da* > *Gal4*; UAS-RFP; UAS-*brat*-RNAi). A significant decrease in the mean growth rate was observed in Passage 2 (p < 0.01, n = 19) and Passage 3 (p < 0.001, n = 8) relative to the first transplantation. (**e**) Cell death was measured in 3rd instar larval brains depleted for Brat (*da* > *Gal4*; UAS-RFP; UAS-*brat*-RNAi) and in serial passages of this tissue. The rate of cell death significantly increased in the second and third passage (p < 0.001, n > 13 for each), but was not significantly different from the larval tissue to the first explant (p > 0.06, n > 13). (**f**) The level of reactive oxygen species (ROS), was measured in labelled 3rd instar larval brains depleted for Brat (*da* > *Gal4*; UAS-RFP; UAS-*brat*-RNAi), and compared with subsequent serial passages of the explanted tissue (P1 to P3) or with tissue treated with an antioxidant (EGCG) or pro-oxidant (Mnd; Menadione) as controls. A strong increase in ROS was observed in the first passage (p < 0.001, n = 10), with a modest increase from passage 1 to passage 3 (p < 0.05, n = 16). (**g**) The rate of chromosomal instability was measured in larval brains (*da* > *Gal4*; UAS-RFP; UAS-*brat*-RNAi) and subsequent passages of this explanted tissue. The proportion of visibly aberrant anaphase figures was significantly higher in the first passage (78 ± 3%, n = 141 compared to 20 ± 3%, n = 622, p < 0.001), but then decreased over the next two passages (p < 0.001, n > 100 for each). (**h,i**) Representative images of normal and defective anaphases from Brat depleted brain tissue (*da* > *Gal4*; UAS-RFP; UAS-*brat*-RNAi), quantitated in (**g**). The arrow indicates an anaphase bridge. In all graphs, error bars show the 95% confidence intervals. Variation in means were tested for significance using Dunnett’s multiple comparisons test. Variation in proportions were tested for significance using Fisher’s exact tests.

Mutations in the gene *brain tumour (brat)* cause tumours in *Drosophila* larval brains due to a failure of neuroblast differentiation^12^, and this tissue can be grown indefinitely as explant tumors if serially transplanted into the abdomen of host adult flies^13^ (Fig. 1a). We initially tested whether depletion of Brat by RNAi gave effective tumour growth comparable to mutant alleles. Dissection of third instar larval brains from control animals marked with RFP and transplanted in to a wild type adult host showed no growth (Fig. 1b), but depletion of Brat by RNAi resulted in strong growth of the RFP-tagged transplanted tissue (Fig. 1c) that would typically kill the host within two weeks. Serial passaging of the tumor tissue after ten days’ growth allowed the development of the tumors to be followed. We observed a significant decrease in the rate of growth (Fig. 1d) and increased cell death (Fig. 1e) in these tumors over the first three passages. Levels of reactive oxygen species (ROS) were high in the explants, with a significant increase relative to the larval brain tumour and from the first to third passage (Fig. 1f). We also observed metastasis, which is a common feature of these explant tumors^14^. Measuring the frequency of aberrant anaphases showed that the CIN rate was 20.2% (±3.2%) in *brat* ^RNAi^ larval brains (n = 15), comparable to our previous CIN model, *mad2* ^RNAi^, which showed defects in 24% of anaphases^3^. We further analysed the CIN rate in *brat* ^RNAi^ tumor explants up to three passages and found the highest CIN rate in passage 1 which decreased over time in the subsequent passages, similar to the explant growth rate (Fig. 1g). These initial studies confirmed that depletion of *brat* by RNAi was a viable method for generating aggressive CIN tumors that shared the high-ROS phenotype that we and others identified when CIN was induced in normal proliferating tissue^7, 15, 16^.

### Effect of metabolic interventions on CIN tumors

**Figure 2:**
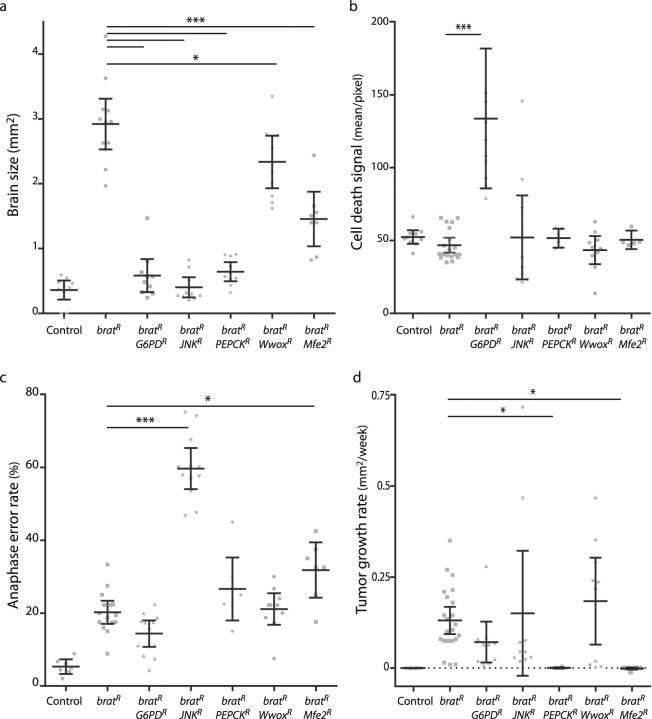
Identifying gene knockdowns that affect CIN tumor growth. (**a**). Comparison of the size of 3rd instar larval brains depleted for Brat (*da* > *Gal4*; UAS-RFP; UAS-*brat*-RNAi) or *brat* and one of five genes known to increase apoptosis in CIN cells. Control brains were *da* > *Gal4*; UAS-RFP. Error bars indicate 95% confidence intervals, n > 10 in all cases. All candidate gene knockdowns gave a significant reduction in larval brain size relative to *brat*-RNAi alone (p < 0.001 for all except *Wwox*-RNAi, p < 0.05). (**b**) Apoptosis in *brat*-RNAi larval brains was compared with *brat* plus candidate knockdowns. Depletion of G6PD showed significantly elevated apoptosis compared to the *brat* alone control (p < 0.001, n ≥10). All other comparisons with the control showed no significant variation (p > 0.05). (**c**) The rate of chromosomal instability was measured in larval brains depleted for Brat (*da* > *Gal4*; UAS-RFP; UAS-*brat*-RNAi) and compared with brains lacking Brat and a candidate. The proportion of aberrant anaphases was elevated relative to the *brat* control (20%, n = 622 anaphases) when JNK (56%, p < 0.001, n = 495) or Mfe2 (32%, p < 0.05, n = 257) were also depleted. All other comparisons with the *brat* control showed no significant variation (p > 0.05 using Fisher’s exact test, n > 200 for each). (**d**) Growth rate of tumor explants. RFP labelled brain tissue from 3rd instar larvae was transplanted into a wild type adult host and growth was measured over two weeks. No growth was observed in control tissue (*da* > *Gal4*; UAS-RFP). Depletion of Brat led to rapid explant growth, which was not significantly affected by co-depleting G6PD, JNK or Wwox (p > 0.05, n ≥ 10). Co-depletion of PEPCK gave a strongly reduced growth rate relative to *brat* alone (p < 0.05, n = 10), as did co-depleting Mfe2 (p < 0.05, n = 10). All error bars show the 95% confidence interval, p values are from Dunnett’s multiple comparisons test except for proportions, for which Fisher’s exact test was used.

**Figure 3:**
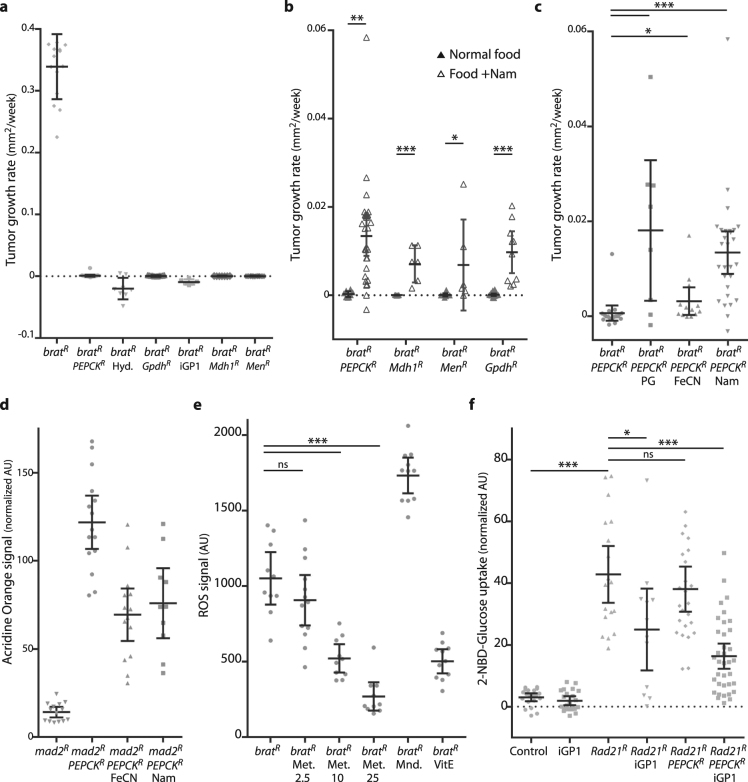
Effect of metabolic intervention on the growth of CIN tumors. (**a**) Labelled 3rd instar brain tissue depleted for Brat (*actin* > *Gal4*; UAS-RFP; UAS-*brat*-RNAi) was grown in wild type hosts for up to two weeks. Feeding the host with the PEPCK inhibitor Hydrazine (Hyd.) blocked tumor growth, as did depleting PEPCK by RNAi. Co-depletion of the cytoplasmic Glycerol-3-Phosphate Dehydrogenase (Gpdh), Malate dehydrogenase (Mdh1) or Malic Enzyme (Men) with Brat was able to block growth, as did feeding the host iGP1 to inhibit the mitochondrial Glycerophosphate Oxidase. All treatments gave a statistically significant decrease in growth relative to the *brat* control (p < 0.001 for each, n ≥ 5) (**b**) The effect of supplementing food with nicotinamide (Nam) was tested on host adults carrying explanted tumors with the indicated genotypes. All genotypes showed significantly increased explant growth when given nicotinamide (multiple t-tests using the Holm-Sidak method, *p < 0.05, **p < 0.01, ***p < 0.001). (**c**) Explants depleted for Brat and PEPCK (*actin* > *Gal4*; UAS-RFP; UAS-*brat*-RNAi; UAS-*PEPCK*-RNAi) did not grow, but could be induced to grow by feeding their adult hosts the antioxidant propyl gallate (PG). Increasing the NAD+/NADH ratio by feeding the hosts ferricyanide (FeCN) or nicotinamide (Nam) could also rescue the explant growth. Significant increases over the *brat PEPCK* control (n = 18) were seen for PG and Nam (p < 0.001, n = 8 and 28), with a modest increase seen for FeCN (p < 0.05 by one-tailed t-test, n = 13). (**d**) The effect of NADH on cell death seen in non-tumorous CIN cells depleted for PEPCK. Cells in the posterior half of 3rd instar larval wing discs depleted for mad2 and PEPCK (*en* > Gal4; UAS-CD8-GFP; UAS-*mad2*-RNAi; UAS-*PEPCK*-RNAi) show a high rate of cell death, as measured by Acridine Orange incorporation^7^, relative to the wild type cells in the anterior half of each disc. The mad2 alone control (en > Gal4; UAS-CD8-GFP; UAS-*mad2*-RNAi; UAS-*LacZ*-RNAi) shows little signal. Increasing the NAD+/NADH ratio by feeding with either ferricyanide (FeCN) or nicotinamide (Nam) significantly decreased the level of cell death (p < 0.001 for both, n ≥ 10). (**e**) The effect of inhibiting the glycerol-3-phosphate shuttle on reactive oxygen species produced in Brat depleted 3rd instar larval brain tissue (*actin* > *Gal4*; UAS-RFP; UAS-*brat*-RNAi). Adding Metformin at 10 mM or 25 mM significantly decreased the level of ROS observed in *brat* tissue (***p < 0.001, n ≥ 10; ns: p > 0.05). Menadione (Mnd.) and vitamin E (VitE) were used as control pro- and anti-oxidant treatments respectively. (**f**) The uptake of labelled glucose in CIN cells was decreased by blocking both PEPCK and the glycerol-3-phosphate shuttle. The posterior halves of 3rd instar larval wing discs were depleted for Rad21 (*en* > Gal4; UAS-CD8-GFP; UAS-*Rad21*-RNAi; UAS-Dicer2) to induce CIN. This strongly increased the uptake of fluorescently labelled 2-NBD-Glucose. Depletion of PEPCK in CIN cells did not significantly decrease the rate of glucose uptake (p > 0.05, n = 25) relative to CIN alone (n = 18). Blocking the glycerol-3-phosphate shuttle by inhibiting GPO1 with iGP1 caused some decrease (p < 0.05, n = 12), while adding iGP to wings depleted for PEPCK strongly decreased the rate of glucose uptake (p < 0.001, n = 36). In all graphs, error bars show the 95% confidence interval. P values given are from Dunnett’s multiple comparisons tests unless otherwise noted.

Having previously shown that cells with induced CIN are sensitive to several metabolic interventions^7^, we now wished to test the effect of such knockdowns on CIN tumors. We tested a range of genes affecting glucose usage (G6PD, PEPCK, Wwox), lipid metabolism (Mfe2) and antioxidant responses (JNK). We initially measured their effect on the size of overgrowth observed in *brat* ^RNAi^larval brains (Fig. 2a). All of the candidate knockdowns tested gave significantly reduced overgrowth at this stage, with some no larger than non-tumorous controls (e.g. *brat* ^RNAi^ *JNK* ^RNAi^). Reduced overgrowth could have been caused by less proliferation or more cell death; our data from wing discs suggested that cell death was likely to be occurring^7^. Surprisingly, we did not observe a significant increase in cell death relative to *brat* alone for any of the candidate knockdowns except Glucose-6-phosphate dehydrogenase (G6PD; Fig. 2b). Elevated CIN rates can also impact proliferation, so we tested whether the knockdown of the candidates was affecting the incidence of CIN in *brat* larval brains. Depletion of Jun N-terminal kinase (JNK) gave a significant increase in the CIN rate, while the others had little (*Mfe2*) or no effect (Fig. 2c). These data indicated that all of the candidates were able to impact the growth of *brat* larval brains, and suggested that varied mechanisms were responsible, with G6PD depletion giving cell death and JNK depletion causing more CIN. When these candidates were tested for their effect on ongoing tumor proliferation in explants, we were surprised to find that neither G6PD nor JNK depletion were able to effectively inhibit *brat* tumor growth (Fig. 2d). However, depletion of either Phosphoenolpyruvate Carboxykinase (PEPCK) or Multifunctional enzyme type 2 (Mfe2) did block the growth of explanted *brat* tumors (Figs 2d and 3).

### The role of NADH in *brat* tumour growth inhibition by PEPCK depletion

**Figure 4:**
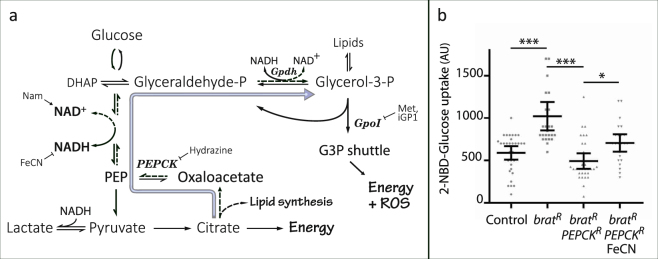
Model for the effect of PEPCK and NADH on glucose metabolism. (**a**) Proliferating cells require glucose for processes including the synthesis of nucleotides (via the pentose-phosphate pathway), membrane lipids (via pyruvate and citrate) and energy (via pyruvate or the glycerol-3-phosphate shuttle). Cytoplasmic NADH is produced in making phosphoenolpyruvate, which must be oxidised for glycolysis to continue. NADH can be oxidised by making lactate, but this prevents the use of pyruvate for lipid synthesis or energy. The glycerol-3-phosphate shuttle can oxidise NADH, but does so at the cost of generating elevated ROS levels. PEPCK mediates glyceroneogenesis (broad arrow), which uses citrate exported from mitochondria to generate glycerol-3-phosphate while oxidising NADH. Our model is that depletion of PEPCK decreases the levels of cytoplasmic NAD+ , which inhibits glycolysis unless the NAD+ can be regenerated by the glycerol-3-phosphate shuttle. This shuttle generates reactive oxygen species, so its use is limited in CIN cells which are already redox stressed. The inhibitors used to test this model are shown with their targets. Abbreviations: DHAP: dihydroxyacetone phosphate, Gpdh: Glycerol-3-phosphate Dehydrogenase, Gpo1: mitochondrial Glycerol-3-Phosphate Dehydrogenase/Glycero-phosphate oxidase, iGP1: inhibitor of Glycerophosphate oxidase 1, Met: Metformin, Nam: nicotinamide, FeCN: ferricyanide, PEPCK: Phosphoenolpyruvate Carboxykinase. (**b**) The effect of PEPCK depletion on glucose uptake in *brat* depleted brain tissue. The uptake of fluorescently labelled 2-NBD-glucose in 3rd instar larval brains was increased by the depletion of Brat (*actin* *>* *Gal4*; UAS-RFP; UAS-*brat-*RNAi; p < 0.001, n ≥ 25). Depleting PEPCK in these *brat* tumors (*actin* > *Gal4*; UAS-RFP; UAS-*brat*-RNAi; UAS-*PEPCK*-RNAi) led to significantly lower uptake of labelled glucose (p < 0.001, n ≥ 25), which was rescued by feeding the larvae with ferricyanide (FeCN, p < 0.05, n ≥ 23). Error bars show 95% confidence intervals. Comparisons were done by multiple t-tests using Tukey’s method.

As an enzyme with a relatively well characterized function in glucose metabolism, PEPCK became the focus of further investigation aimed at explaining how CIN tumor growth can be blocked. We found that feeding the hosts an inhibitor to PEPCK (hydrazine; Hyd) was able to block *brat* explant growth, consistent with our depletion of PEPCK in the tumor by RNAi (Fig. 3). PEPCK catalyses the inter-conversion of oxaloacetate and phosphoenolpyruvate (Fig. 4a) and is rate limiting for gluco- and glyceroneogenesis^17, 18^. Proliferating cells must generate lipids and nucleotides, and to do so typically drive glycolysis at a high rate. This leads to a build-up of NADH in the cytoplasm which must be cleared for glycolysis to continue. The glycerol-3-phosphate shuttle is an important sink for NADH that could be significant for the growth of *brat* tumors^19^. PEPCK is required for glyceroneogenesis (Fig. 4a), which is a significant contributor to the generation of glycerol-3-phosphate, even in the presence of glucose^20^. We tested the requirement for this shuttle by either depleting cytoplasmic Glycerol-3-Phosphate Dehydrogenase (Gpdh) in the tumour or by feeding the host a specific inhibitor (iGP1) of GPO1, the mitochondrial Glycerophosphate Oxidase^19^. In either case, the growth of *brat* explants was blocked (Fig. 3a). We were not able to block growth by inhibiting hexokinase or uncoupling mitochondrial respiration (data not shown). The inhibition of tumor growth by blocking the glycerol phosphate shuttle suggested that these tumors may be sensitive to cytoplasmic NADH build-up.

### Replacing NAD + can rescue *PEPCK brat* explant growth

**Figure 5:**
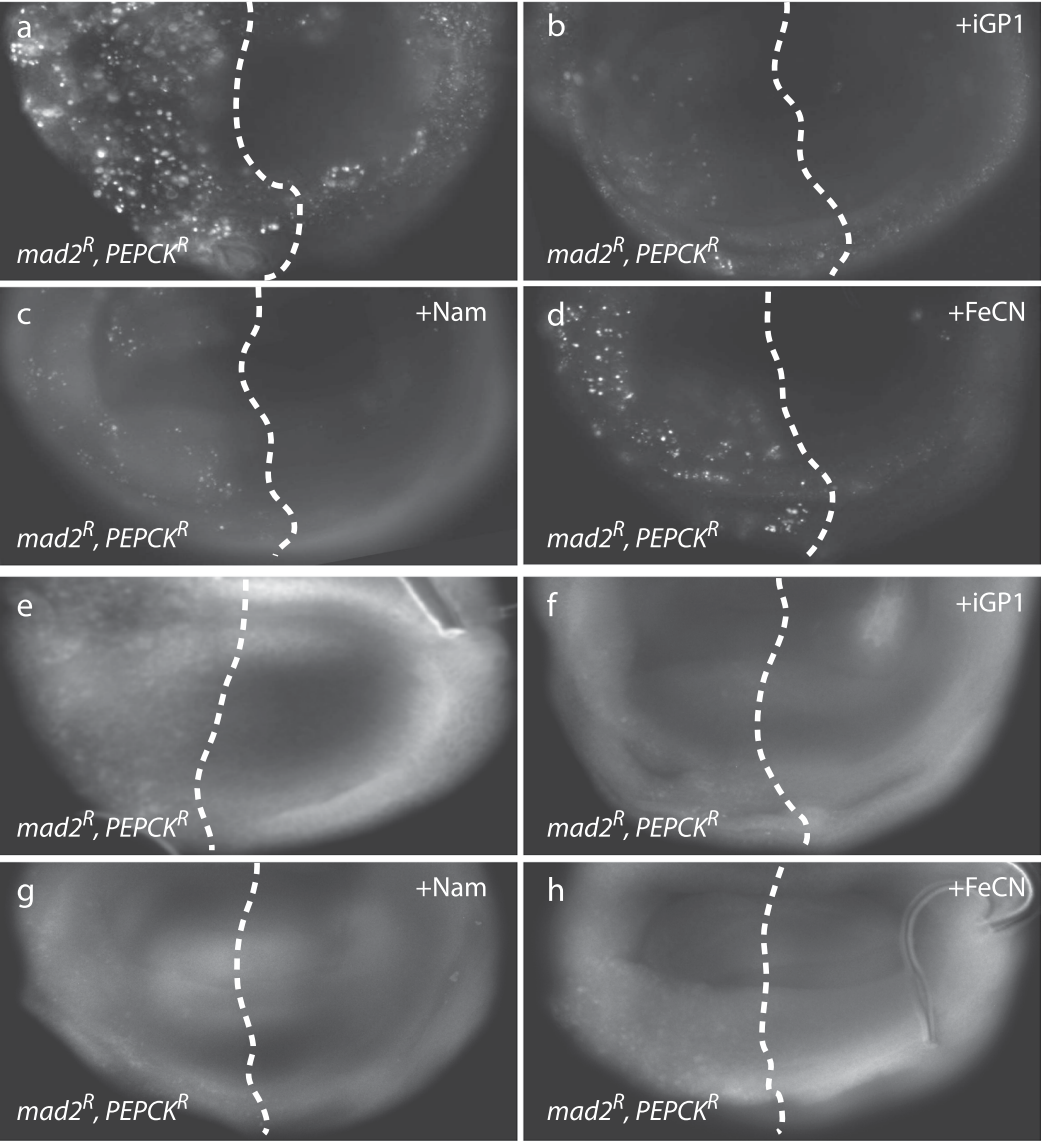
The effect of cytoplasmic NADH levels on the survival and metabolism of CIN cells depleted for PEPCK. (**a**–**d**) Cell death caused by depletion of Mad2 and PEPCK could be rescued by feeding larvae chemicals to alter cytoplasmic NADH levels. (**a**) Cells in the posterior half of 3^rd^ instar larval wing discs (left of the dotted lines) were depleted for Mad2 and PEPCK (*en* > Gal4; UAS-CD8-GFP; UAS-*mad2*-RNAi; UAS-*PEPCK*-RNAi) and showed an elevated level of cell death, as visualized by Acridine Orange^7^, relative to the wild type cells in the anterior half of each disc. Increasing the cytoplasmic NAD+/NADH ratio by feeding with either ferricyanide (**d**, FeCN) or nicotinamide (**c**, Nam) significantly decreased the level of Acridine staining, as did blocking the glycerol phosphate shuttle by feeding iGP1 (**b**). Quantitation of the rescue is shown in Fig. 3d. (**e**–**h**) ROS caused by depletion of Mad2 and PEPCK could also be rescued by changing NADH availability. (**e**) Cells in the posterior half of 3rd instar larval wing discs (left of the dotted lines) were depleted for Mad2 and PEPCK (*en* > Gal4; UAS-CD8-GFP; UAS-*mad2*-RNAi; UAS-*PEPCK*-RNAi) and showed an elevated level of ROS, as visualized by CellRox, relative to the wild type cells in the anterior half of each disc. Increasing the cytoplasmic NAD+/NADH ratio by feeding with either 5 mM ferricyanide (h, FeCN) or 5 mM nicotinamide (g, Nam) decreased the level of CellRox staining, as did blocking the glycerol phosphate shuttle by feeding with 2 μM iGP1 (**f**).

This model for the role of NADH in mediating the effect of PEPCK on *brat* tumor explants would predict that providing the tumor with NAD+ should bypass the need for PEPCK, allowing glycolysis to proceed and *PEPCK brat* explants to grow. We tested this model by feeding the host adult nicotinamide (Nam), a precursor in the synthesis of NAD+ ^21^. While *brat* tumors depleted for PEPCK did not grow, we found that the same tumors in hosts fed nicotinamide were able to grow, albeit slowly (Fig. 3b). This strongly suggested that lack of NAD+ was a limiting factor when PEPCK was depleted. We also found that nicotinamide rescued the growth of *brat* tumors lacking Gpdh (Fig. 3b), confirming the importance of the glycerol-3-phosphate shuttle in oxidising NADH in these tumors. Ideally we would have confirmed this by measuring cytoplasmic NAD+/NADH ratios in the explants, but the low NADH concentration combined with tiny tissue size made this technically problematic. As an alternative approach, we confirmed that cytoplasmic NADH was growth limiting in PEPCK depleted tumors by feeding the hosts ferricyanide (FeCN)^22^ which externally drives NADH oxidation, and this was also able to increase growth (Fig. 3c). To test whether this rescue of *PEPCK* phenotypes by NADH oxidation was a feature only of *brat* tumors, we tested non-tumorous proliferating cells in which CIN had been induced by Mad2 depletion. The level of cell death and ROS induced by PEPCK depletion in this CIN tissue was significantly rescued by feeding the larvae either ferricyanide or nicotinamide (Figs 3d and 5). Proliferating wing disc tissue in which CIN was induced by BubR1 depletion showed similar sensitivity to PEPCK depletion (Supplementary Figure 2).

An important side-effect of using the glycerol-3-phosphate shuttle is the production of ROS by mitochondrial Glycerophosphate Oxidase (GPO1)^23, 24^. Given that we observed high levels of ROS in *brat* tumor explants (Fig. 1f), we wished to test whether ROS levels were limiting the usefulness of the glycerol-3-phosphate shuttle when PEPCK was depleted. When adult hosts were fed with an antioxidant (propyl gallate; PG), we observed a significant rescue of the growth of PEPCK depleted *brat* tumor explants (Fig. 3c). We tested whether the antioxidant rescue seen with propyl gallate was affecting ROS generated by the glycerol phosphate shuttle, by feeding Brat depleted larvae metformin, which inhibits mitochondrial Glycerol-3-phosphate Dehydrogenase/GPO1^25^ (Fig. 3e). Increasing levels of metformin gave decreasing levels of ROS, consistent with the ROS in *brat-*RNAi brain tissue being generated by the glycerol-3-phosphate shuttle. Similarly, adding a specific inhibitor to the glycerol phosphate shuttle in cells depleted for PEPCK and Mad2 also lowered ROS levels (Fig. 5f).

CIN tissues can have an elevated metabolic rate, consuming energy stores faster and producing more mitochondrial output than normal proliferating cells^7^. This was reflected in an increased rate of uptake of labelled glucose in proliferating CIN tissue (*Rad21*-RNAi) relative to controls (Fig. 3f). Feeding the animals a Gpo1 inhibitor to block the glycerol-3-phosphate shuttle significantly decreased the uptake of glucose (Fig. 3f), consistent with this shuttle being an important sink for NADH that allows glycolysis to continue at a high rate. Depletion of PEPCK did not strongly block glucose uptake (Supplementary Figure 3), however in *brat* tumors, depletion of PEPCK had a stronger effect (Fig. 4b), and could be rescued by feeding ferricyanide, confirming the importance of NADH oxidation for continued glycolysis in these tumors.

## Discussion

CIN induced in *Drosophila* wing discs has reproducible phenotypic effects^3–5, 15, 26^, even though the genetic instability is effectively random. CIN cells typically generate reactive oxygen species, activate the p38 and JNK/Upd pathways, drop out of the epithelial layer and trigger an innate immune response. Because of this stereotypical response to induced aneuploidy, we reasoned that there should be ways to effectively target the response that should be specific to CIN cells. By screening for such CIN-killing interventions, we found several, such as targeting JNK or centrosomes, that were effective but not ideal due to their important roles in normal proliferating cells^3^. Metabolic intervention, on the other hand, has better potential as a therapeutic tool because normal cells can tolerate large changes in metabolite concentrations, which they experience during feeding and fasting. On that basis we wished to further investigate the effect of metabolic intervention not just on epithelial cells with induced CIN, but on a CIN tumour growing *in vivo*.

Explanted *Drosophila* brain tumours have been used for over a decade^13^ and allow the development of a tumour to be followed for months by regular passaging. These tumours have been shown to accumulate additional centrosomes and become aneuploid, so we were not surprised to find that the CIN rate in transplanted *brat* tissue was relatively high. We also observed an elevated level of reactive oxygen species, consistent with previous findings from *Drosophila* cells with induced CIN^7, 15^ and from stable aneuploidy in a range of organisms^27^. Of course, *brat* tumours have other changes in addition to CIN^28^, but the phenotype of the *brat* tumours was sufficiently similar to our induced-CIN model to encourage us to examine the best metabolic candidates we had identified in the wing disc CIN model. Surprisingly, depleting proteins like G6PD or JNK had little effect on tumour explant growth (Fig. 2d), though they both strongly induced cell death in our wing disc model^3^. We considered whether the number of unstable divisions during the extended proliferation period available to the explants might have generated sufficient variation to allow the appearance of mutant clones resistant to the effect of our knockdowns. We think this unlikely, as other similar interventions, such as *PEPCK*-RNAi never acquired resistance and for *G6PD* or *JNK* we did not see a period of limited growth followed by rapid expansion, which would be expected if tumours were acquiring resistance. Instead we saw immediate growth upon transplantation, suggesting that in this environment, those knockdowns were not able to effectively block cell proliferation. Prior to transplantation, knockdown of *G6PD* did give reduced brain size and increased ROS and cell death in *brat* larval brains, suggesting that some feature of the transplantation environment was able to rescue their growth. Further investigation will be needed to account for this growth difference.

Following our initial tests, we focused our attention on *PEPCK*, which consistently blocked the growth of explanted tumours. PEPCK is best known for its role in the liver, where it mediates gluconeogenesis during fasting^29^. However, PEPCK is expressed widely in non-gluconeogenic tissue in most organisms, notably in muscle and gut as well as cancer cells, where its role is more complex, facilitating the catabolism of either glucose or glutamine^30^. PEPCK is regulated transcriptionally by p38 via ATF-2^31^ and post-translationally by acetylation^32^. PEPCK is rate limiting for glyceroneogenesis, the synthesis of glycerol-3-phosphate from TCA cycle intermediates^33^. This is particularly important for the re-esterification of free fatty acids, which is needed to prevent the depletion of fats that we and others have noted when PEPCK is removed^7, 34^. Cell proliferation requires the doubling of membrane lipids at each division, so this demand for lipid synthesis could be an important role for PEPCK in non-gluconeogenic tissues. However, *PEPCK* null mutants and RNAi knockdowns remain viable^3, 34^, so this is insufficient to explain why *PEPCK* depleted *brat* explants cannot grow and why *PEPCK* depleted CIN wing disc cells apoptose. A possible explanation is suggested by our observation that providing antioxidants or increasing NAD+ could rescue the growth of *PEPCK* depleted *brat* explants. Our data suggest that these interventions affected the glycerol phosphate shuttle, which may be an important sink for the NADH generated during glycolysis, but which also generates ROS^23^.

NADH is made from NAD+ in the cytoplasm during glycolysis, and must be oxidised for glycolysis to continue. Pyruvate can be converted to lactate to regenerate NAD+ , however this option is limited in proliferative cells as pyruvate is in demand for energy and lipid synthesis (Fig. 4a). In some organisms the Malate/Aspartate shuttle is used to lower levels of cytoplasmic NADH, however in *Drosophila*, the Mdh1 and Got1 enzymes that would catalyse the cytoplasmic half of the shuttle are found in peroxisomes^35^. Nonetheless, our data suggest Mdh1 is still needed to regulate NADH in CIN tumors (Fig. 3a,b). An effective alternative for oxidising cytoplasmic NADH is via the glycerol-phosphate shuttle. This shuttle uses Glycerol Phosphate Dehydrogenase to divert from glycolysis as much glyceraldehyde phosphate as required to oxidise NADH. This makes glycerol-3-phosphate, which can be removed by dephosphorylation to glycerol, or by acylation to make fats, however the shuttle uses mitochondrial Glycerophosphate Oxidase to generate energy in the mitochondria while recovering the glyceraldehyde phosphate for glycolysis. This pathway is heavily used in muscle, but it can also generate significant levels of ROS^23^. We found that the shuttle is important in proliferating CIN cells, as inhibiting or depleting Glycerophosphate Oxidase caused a significant decrease in glucose uptake, and blocked the growth of *brat* explants.

We found that increasing the availability of NAD+ by feeding the host nicotinamide rescued the growth of PEPCK depleted *brat* explants, suggesting that in these tumours, the glycerol phosphate shuttle has not been able to sufficiently oxidise NADH, despite the availability of glucose to fuel this pathway. One possible contributor to this limitation was the ROS generated by mitochondrial Glycerophosphate Oxidase (GPO1)^23^, because we know that cells with CIN are already redox stressed^7^. Consistent with this model (Fig. 4a), we found that inhibiting Glycerophosphate Oxidase lowered ROS levels but did not allow growth of *brat* explants (as the shuttle was then unavailable for NADH oxidation). However, lowering ROS by feeding the host antioxidants was able to rescue growth: in this case the shuttle is available without the toxic consequences of high ROS. Lowering endogenous antioxidants by depleting Malic enzyme (hence NADPH)^36^ was able to block the growth of *brat* explants. This effect of ROS levels on PEPCK depleted explants suggests that the glycerol-3-phophate formed in glyceroneogenesis is not just used for lipids, but is also used to clear cytoplasmic NADH via the mitochondria. It is still poorly understood how PEPCK mediates retrograde carbon flow in the presence of glucose, but high rates of PEPCK-mediated glyceroneogenesis are observed in numerous cell types and diets, including cancer cells^18, 20, 37, 38^.

Our model, then, for effectively blocking the growth of *brat* tumours is that treatments that decrease the use of the glycerol-3-phosphate shuttle (*PEPCK-*RNAi, hydrazine, *GPDH-*RNAi, iGP1) are inhibiting growth by preventing the oxidation of cytoplasmic NADH. If the shuttle is available, its activity is significantly limited by the production of ROS in redox stressed CIN cells, so treatments that lower ROS levels or that provide an alternative source of NAD+ , will tend to rescue tumour growth. By using the appropriate level of PEPCK and GPO1 inhibitors we could completely block the growth of these aggressive CIN tumours.

These results emphasize the benefit of starting with an unbiased genetic approach to identifying the key sensitivities of CIN cells. We would not have predicted that PEPCK had a significant role in managing NADH, nor that this would be enough to block tumour growth *in vivo*. It is not surprising that ROS levels can limit tumour growth, as they provide a mutagenic advantage to tumours only at the cost of widespread cellular damage. We had not previously considered mitochondrial Glycerophosphate Oxidase as a significant source of ROS, however we find that in *PEPCK brat* tumors, the glycerol-3-phosphate shuttle generates growth limiting ROS levels. Blocking this pathway lowers ROS levels, but it also prevents PEPCK-inhibited tumours from oxidising NADH, which then becomes growth limiting. This trade-off between NADH and ROS suggests that the relationship between the glycerol-phosphate shuttle and NADH sources may be a fruitful area of research for combination therapy directed at CIN tumors.

### Experimental procedures

#### Oxidative stress analysis

Oxidative damage was analysed in tumor tissue using CellRox Green (Invitrogen) according to the manufacturers’ recommendations. Menadione (10 mM), and Vitamin E (5 mM) in D22 media were used as a pro-oxidant positive control and anti-oxidant control. Another anti-oxidant epigallocatechin gallate-EGCG (10 µM) was used in some experiments. Tissue was dissected in D22 media then stained in the ROS dye for 20 minutes. Photographs were taken on a Zeiss Axioplan 2 using a fixed exposure time for each experiment determined by the positive control (Menadione) treated tissue.

Passaged *brat* ^*RNAi*^ tissue was dissected and incubated for ROS staining in fly extract media. Fly extract was prepared using Schneider’s *Drosophila* media (0.93 ml), whole fly extract of 200 fly (50 µl), insulin 0.5 mg/1 ml (5 µl), penicillin/streptomycin 10,000U/ml (5 µl)^39^.

#### Glucose assay

A labelled fluorescent glucose, 2-NBDG (Sigma) was used to detect the uptake of glucose as recommended by the supplier. Wing discs were taken from 3rd instar larvae that were still feeding to ensure glycolytic metabolism and immediately incubated in 2 μM 2-NBDG for 1.5 h in wing disc culture media^39^ before imaging. Images were taken at 20X with a fixed exposure time for all genotypes and fluorescent intensity was measured by using ImageJ software.

#### Explant and measurement techniques

An injection system was developed using 1.0mm O.D × 0.78mm I.D. borosilicate glass capillaries, a capillary holder, a suction tube and a suction apparatus. All the explants were done under a dissection microscope, into the ventral side of the fly abdomen^13, 40^. RFP was used to detect the explant presence and its growth. All explants were photographed at 3X under a fluorescence dissecting microscope (Nikon SMZ1500). The number of hosts that survived the transplant and hence contributed to the results for each experiment is shown by the number of points on its respective graph.

#### CIN analysis

CIN was analysed by measuring the rate of anaphase errors. Whole larval brains were fixed using 3.7% formaldehyde in PBS for 20 minutes, put in 45% and 60% acetic acid in ddH_2_O for 2 minutes and 45 seconds respectively, then squashed onto a cover slip and frozen in liquid nitrogen. Hoechst 33342 (Sigma) at 2 μg/ml in PBS was used to stain chromosomes. 50 anaphases were identified per brain and anaphase aberrations were scored. CIN analysis for passaged tissue was treated similarly, except all the available anaphases of each explant were scored, due to the smaller amount of tissue.

#### Cell death analysis

Brain: whole brains were incubated in CellEvent Caspase 3/7 Green (Thermo Fisher) according to the recommended protocol. After the treatment, brains were mounted and photographed at 10X on a Zeiss Axioplan 2. GFP signals were counted using the Analyse Particle plugin in ImageJ. Passaged tissues were treated in a similar way, except the signal was manually counted using the Cell Counter plugin.

Wing discs: wing discs were treated with 1 mM Acridine Orange/PBS stain for three minutes, then briefly washed. The discs were mounted in PBS and photographed at 10X on a Zeiss Axoplan 2. The intensity of RFP signal was measured in ImageJ and normalized relative to the control half of the disc as described^3^. For ROS analysis of wing discs we used CellRox Deep Red (Invitrogen) as described [7].

#### Drug treatments

Drugs were obtained from Sigma except where noted. For adult fly feeding, drugs were mixed in 20% sucrose solution. For larvae, drugs were mixed in standard fly food (water, molasses, yeast, glucose, acid-mix, agar, semolina, Tegosept) and were given to the host fly when solidified. Drugs used were as follows unless otherwise noted in figure legends: iGP1 (Vitas-M lab, 1 mM), hydrazine sulphate-HS (10 mM), Metformin-Met (25 mM), Ferricyanide-FeCN (0.05 mM), Nicotinamide-Nam (5 mM), Propyl gallate-PG (1 mM). Because hydrazine and metformin are relatively non-specific in their targets, they were only used in cases where we could verify the relevance of the phenotype by comparison with the RNAi phenotype of the relevant enzyme (PEPCK or GPO1).

#### Drosophila stocks

Fly stocks were from either the Vienna *Drosophila* Resource Centre or Bloomington Stock Centre, raised at 25 °C, and all treatments were done at room temperature. Gal4 drivers included *daughterless-*gal4 and *actin*-gal4 for brain expression and *engrailed*-gal4 for wing expression of UAS transgenes. UAS-*RFP* was used as a reporter gene in *actin*-Gal4 and *da*-Gal4 driven UAS-*brat* ^*RNAi*^ tumours. Canton S female flies were used as hosts for the CIN tumor explants. Stocks used were as follows; UAS-*brat* ^*RNAi*^ (34646), UAS-RFP (27391), UAS-*Mfe2* ^*RNAi*^ (v108880), UAS-*PEPCK* ^*RNAi*^ (v20529), UAS-*G6PD* ^*RNAi*^ (v101507), UAS-*JNK* ^*RNAi*^ (v34138), UAS-*Wwox* ^*RNAi*^ (v108307), UAS-*Men* ^*RNAi*^ (41652), UAS-*Gpdh* ^*RNAi*^ (v29013/GD), UAS-*Mdh1* ^*RNAi*^ (v41437/GD), UAS-*mad2* ^*RNAi*^ (v47918), UAS-*rad21* ^*RNAi*^ (v13669), UAS-*dicer2*. Standard crosses were used to generate the genotypes tested, using segregation away from *Bl*/*CyO*;*TM2/TM6b* to combine markers on 2nd and 3rd chromosomes. Where recombination was necessary (*en *>* Gal4 UAS-mad2*), the stock was tested for each locus and taken through single pair matings to ensure a consistent genotype. The level of depletion by RNAi shown in Supplementary Table 1 was measured by qPCR as described (7) using the following primers:

*Pepck*: (f) CCGTGTGCTGGAATGGATC (r) TTGGGCAGCGAGAAGATCT;

*brat*: (f) AACCACAACAACTTCAACCTGAC (r) GCGATATATGTAGAGCCGATAGTC;

*Gpdh*: (f) TCACGACGTGTTACGGTGG (r) CCTCAATGGTTTTTCCAGAAGT;

*rp49* control (f) GACGCTTCAAGGGACAGTATCTG (r) AAACGCGGTTCTGCATGAG.

### Statistical analysis

In most cases, Dunnett’s multiple comparisons test was used to assess the significance of variation in the means of each genotype of test tumour from that observed in the control. A single-tailed t-test with Welch’s correction was also used for testing growth relative to a non-growing control. This was only used in one case of doubtful significance (the effect of FeCN on *brat PEPCK* tumor growth), which was also confirmed by other tests (e.g. cell death rescue and the effect of nicotinamide). Fisher’s exact tests were used to compare proportions (CIN rates). In all graphs, error bars show 95% confidence intervals.

### Data availability

All data generated or analysed during this study are included in this published article (and its Supplementary Information files).

## Electronic supplementary material

Supplementary Information
