# Supplementary material for: Phosphoenolpyruvate Carboxykinase Maintains Glycolysis-driven Growth in Drosophila Tumors
Source: Sci Rep. 2017 Sep 14;7:11531. doi: 10.1038/s41598-017-11613-2 (PMC5599506; doi:10.1038/s41598-017-11613-2)
Supplement: Supplementary file 1 — Supplementary Information [file 41598_2017_11613_MOESM1_ESM.pdf]

Phosphoenolpyruvate Carboxykinase Maintains Glycolysis-driven Growth in Drosophila Tumors.

Rashid Hussain, Zeeshan Shaukat, Mahwish Khan, Robert Saint and Stephen L. Gregory.

Supplementary Information.

Supplementary Figure 1

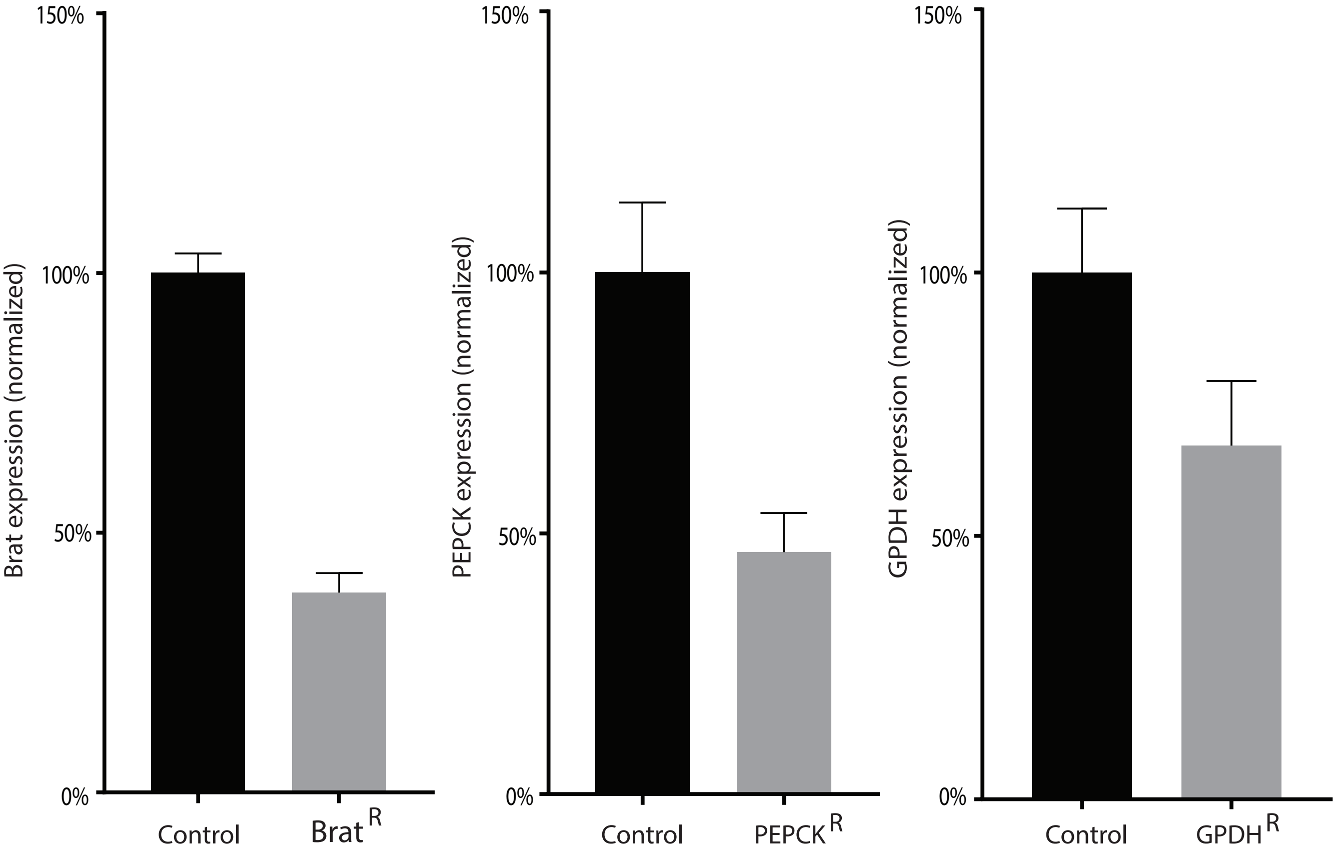

Supplementary Figure 2

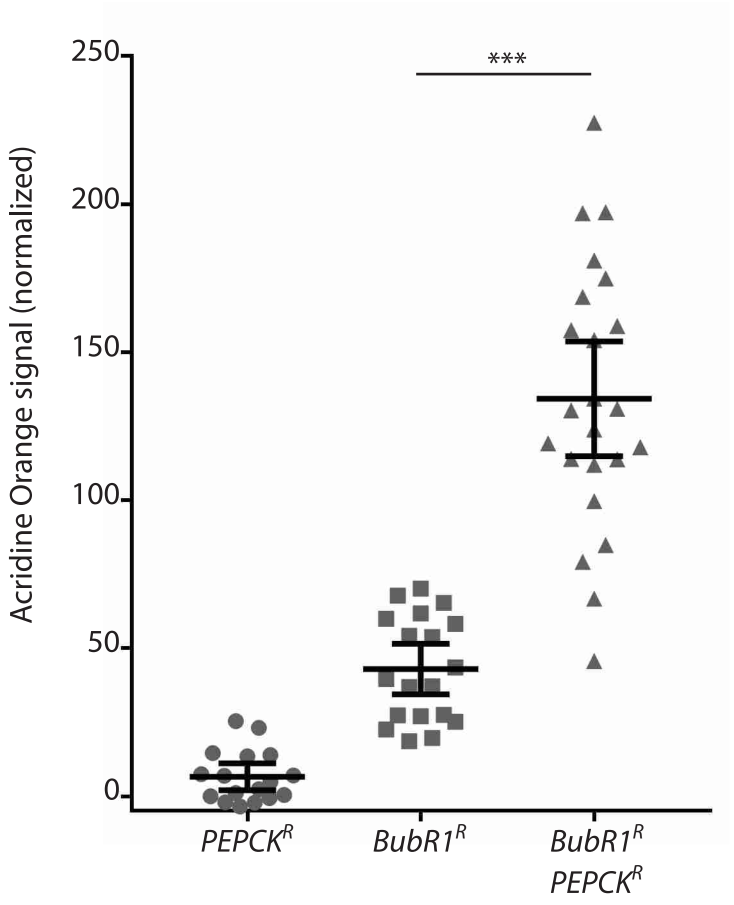

Effect of PEPCK depletion on cell death levels in Drosophila imaginal wing disc cells when CIN was induced by the depletion of BubR1. Depletion of BubR1 to induce CIN in the posterior half of Drosophila 3rd instar larval wing discs (*en*>*Gal4;* UAS-*BubR1*-RNAi) gave some cell death, which was strongly enhanced by co-depleting PEPCK (*en*>*Gal4;* UAS-*BubR1*-RNAi; UAS-*PEPCK*-RNAi). Error bars show 95% confidence intervals, *** indicates $p<0.001$ by unpaired t-test.

Supplementary figure 3

a b

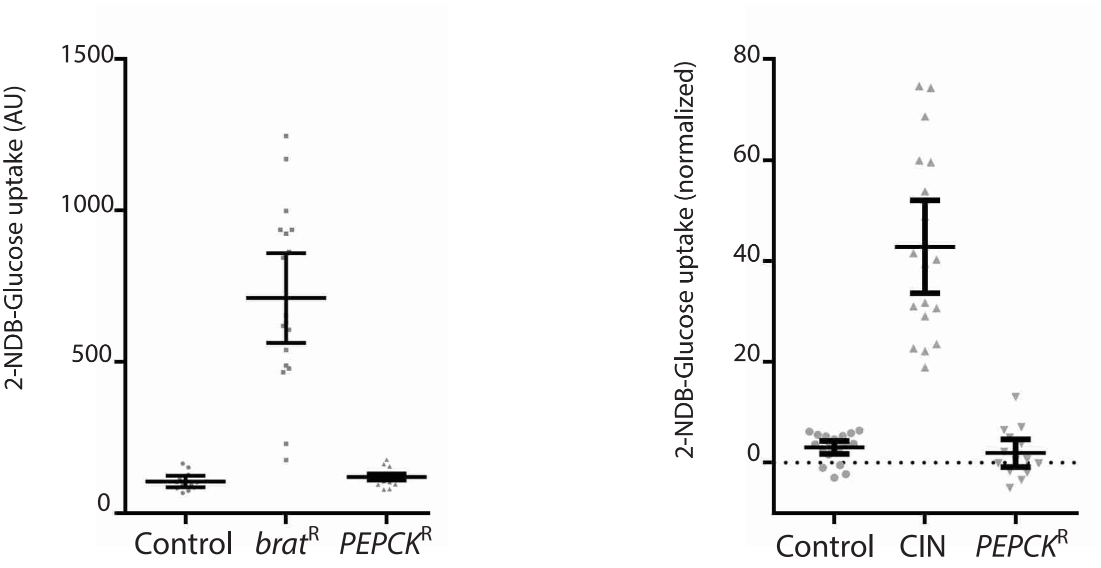

Depletion of PEPCK alone does not affect glucose uptake. (a) The uptake of fluorescently labelled 2-NDB-glucose in 3rd instar larval brains was increased by depletion of Brat (*da*>*Gal4*; UAS-*brat*-RNAi), but not altered by depletion of PEPCK (*da*>*Gal4*; UAS-*PEPCK*-RNAi). (b) The uptake of fluorescently labelled 2-NDB-glucose in the posterior half of 3rd instar larval wing discs was increased when CIN was induced by depletion of Rad21 (*en*>*Gal4*; UAS-*Rad21*-RNAi; UAS-*Dicer2*), but not altered by depletion of PEPCK (*en*>*Gal4*; UAS-*PEPCK*-RNAi).
